# Inoculation of *Cenococcum geophilum* enhances heat tolerance in *Pinus massoniana* through integrated physiological, biochemical, and transcriptional reprogramming

**DOI:** 10.1128/aem.02491-25

**Published:** 2026-07-10

**Authors:** Taoxiang Zhang, Yijia Zhou, Shihuan Zhong, Yihang Yang, Wenbo Pang, Feng Cai, Huaihai Chen

**Affiliations:** 1 International Joint Laboratory of Forest Symbiology, College of Forestry, Fujian Agriculture and Forestry University 12449, Fuzhou, Fujian, China; 2 State Key Laboratory of Biocontrol, School of Ecology, Shenzhen Campus of Sun Yat-sen University 582261, Shenzhen, China; The University of Arizona, Tucson, Arizona, USA

**Keywords:** *Pinus massoniana*, ectomycorrhizal inoculation, thermotolerance, nitric oxide (NO), proline metabolism, transcriptomic regulation

## Abstract

Global warming is increasing the frequency of extreme heat events, threatening forest resilience. However, the physiological and molecular mechanisms underlying heat tolerance of ectomycorrhizal (ECM) inoculation in conifers remain poorly understood. This study investigates how the ECM fungus *Cenococcum geophilum* enhances thermotolerance in *Pinus massoniana* seedlings. We found that ECM inoculation significantly improved plant biomass and photosynthesis under both normal and high temperatures. Under heat stress, ECM symbiosis reduced oxidative damage by elevating the activities of antioxidant enzymes (superoxide dismutase [SOD], catalase [CAT], and peroxidase [POD]) and promoting nitric oxide (NO) accumulation via enhanced nitrate reductase (NR)- and nitric oxide synthase (NOS)-dependent pathways. Furthermore, ECM colonization reprogrammed proline metabolism, stimulating its biosynthesis through Δ1-pyrroline-5-carboxylate synthetase (P5CS) and ornithine aminotransferase (OAT) while tissue-specifically regulating proline dehydrogenase (ProDH), thereby supporting osmotic adjustment in roots and energy maintenance in shoots. Transcriptomic analyses revealed that ECM primed the host at 25°C by activating defense signaling, reinforcing epidermal structures, and enhancing starch and sucrose metabolism. Under heat stress, ECM induced extensive transcriptional reorganization, upregulating pathways related to membrane lipid remodeling, cell wall modification, and carbon reallocation, while downregulating energy-costly processes, such as oxidative phosphorylation and RNA polymerase activity. This shift reflects an ECM-driven resource reallocation strategy that suppresses ROS production and prioritizes cellular integrity. Collectively, our study demonstrates that *C. geophilum* establishes an integrated mechanism involving physiological, biochemical, and transcriptional adjustments to enhance heat tolerance in *P. massoniana*, providing mechanistic insight into ECM-mediated climate resilience and underscoring the potential of using ECM fungi as an ecological tool to promote forest adaptation in a warming world.

**IMPORTANCE:**

This study elucidates how the ectomycorrhizal fungus *Cenococcum geophilum* systemically enhances heat tolerance in *Pinus massoniana* by acting as a natural “heat shield,” revealing a symbiotic mechanism where the fungus primes the plant's antioxidant defenses, reprograms proline metabolism in a tissue-specific manner, and boosts nitric oxide signaling. Crucially, transcriptomic analysis shows the fungus drives a strategic resource reallocation under heat stress by upregulating pathways for cellular integrity while downregulating energy-intensive processes to minimize oxidative damage. These findings establish a detailed mechanistic framework for fungal-mediated climate resilience, highlighting that enhancing natural partnerships with soil fungi can fortify existing forests against increasing heatwaves by optimizing internal stress management for forest adaptation, offering a practical tool for ecosystem management in a warming world.

## INTRODUCTION

Global warming, driven by escalating atmospheric CO_2_ levels, is projected to increase global temperatures by 2.4–6.4°C by the end of this century (1). This trend poses a substantial threat to forestry production and ecological security worldwide. In particular, the increasing frequency and severity of heat stress events have already caused widespread forest mortality in regions of Southern Europe, North America, and subtropical Asia, primarily through the disruption of key plant physiological processes of photosynthesis, nutrient uptake, and redox balance (2–4). To mitigate these risks, the identification and cultivation of heat-tolerant tree species have become imperative for sustaining forest productivity and ecosystem stability. In this context, Masson pine (*Pinus massoniana*), a widely distributed afforestation species in subtropical China, has drawn significant attention as a tree of high ecological and economic value, prized for its adaptability, fast growth, and high yields (5). However, studies have documented that under elevated temperatures and drought, *P. massoniana* exhibits marked reductions in photosynthetic efficiency, disruption in osmotic regulation, and suppression of biomass accumulation (6, 7). This growth suppression under elevated temperature is not unique to *P. massoniana*; high temperatures universally impair plant physiological processes, reducing biomass accumulation and compromising stress tolerance across species. Therefore, investigating the physiological mechanisms underlying these heat-induced limitations and developing targeted strategies to enhance their thermal tolerance are crucial for ensuring the future health and sustainability of forests dominated by this species in the context of global warming.

To enhance tree thermotolerance, mycorrhizal fungi, which form mutualistic symbiotic relationships with the majority of trees and play a significant role in plant growth, succession, and adaptation to adverse conditions, present a promising biological solution. Notably, they are known to enhance the host plant’s tolerance to temperature stress through multiple pathways, including improving water and nutrient acquisition, enhancing photosynthesis, activating antioxidant systems, promoting the accumulation of osmolytes like proline, and facilitating the secretion of signaling molecules (8). At the physiological level, ectomycorrhizal (ECM) symbiosis enhances osmotic adjustment by stimulating proline biosynthesis via key enzymes such as Δ1-pyrroline-5-carboxylate synthetase (P5CS) and ornithine aminotransferase (OAT) while tissue-specifically modulating proline dehydrogenase (ProDH) to balance osmoprotection and energy supply (9, 10). At the metabolic level, ECM fungi reshape host nitrogen metabolism by enhancing nitrate acquisition and assimilation, sustaining the biosynthesis of osmoprotectants and antioxidant compounds essential for thermotolerance (11, 12). Functionally, the extensive extraradical mycelium, along with fungal metabolites and extracellular enzymes, not only increases soil nutrient availability but also critically enhances the host plant’s ability to absorb and transport nutrients under stress conditions, thereby improving host plant biomass and stress tolerance (13, 14). Furthermore, under high temperatures, mycorrhizal symbiosis helps mitigate oxidative damage by upregulating key antioxidant enzymes, including superoxide dismutase (SOD), peroxidase (POD), and catalase (CAT), which reduce reactive oxygen species (ROS) accumulation (8, 15). Given this multi-functional role, the strategic inoculation of mycorrhizal fungi emerges as a highly promising and ecologically sustainable approach to fortifying trees against the escalating threats of global warming.

Among the key signaling molecules regulating symbiosis, proline is a crucial osmolyte enabling plants to cope with heat stress by regulating osmotic balance, reducing water loss, and protecting cell membrane integrity, primarily via the glutamate and ornithine pathways (16, 17). Notably, mycorrhizal colonization has been demonstrated to significantly promote proline accumulation in host plants, directly contributing to enhanced heat tolerance (6). Another critical component is nitric oxide (NO), a signaling molecule that acts synergistically with mycorrhizal symbiosis to alleviate oxidative damage and regulate the expression of stress-responsive genes (8, 18, 19). Under heat stress specifically, NO accumulation has been shown to counteract ROS overproduction and activate antioxidant defense systems primarily through the nitrate reductase (NR)- and nitric oxide synthase (NOS)-dependent pathways, thereby stabilizing redox homeostasis and enhancing plant stress tolerance (18, 20). Critically, mycorrhizal colonization has been identified as a key driver of host NO biosynthesis by enhancing nitrate acquisition and upregulating NR and NOS activities, which in turn amplifies antioxidant gene expression and enzyme activities, and stimulates the accumulation of protective osmolytes such as proline, ultimately alleviating stress-induced growth inhibition (21, 22). While mycorrhizal symbiosis is recognized as an effective ecological strategy to enhance forest resilience under climate change, critical knowledge gaps persist regarding its regulatory effects on proline accumulation and metabolism, as well as the underpinnings of NO-mediated thermotolerance, both of which crucially involve enzyme-mediated mechanisms.

Against this backdrop, the ectomycorrhizal (ECM) fungus *Cenococcum geophilum* emerges as a highly pertinent model for investigation. As a prevalent symbiont in forest ecosystems, it associates with a wide range of tree hosts and demonstrates remarkable adaptability to both biotic and abiotic stresses (13, 23). Notably, *C. geophilum* harbors exceptionally high intraspecific genetic diversity and is increasingly recognized as a species complex comprising multiple cryptic lineages with distinct ecological traits and stress-response profiles, distributed across diverse biomes from boreal to tropical forests (24). This genetic variability is particularly relevant under stress conditions, as different ecotypes may exhibit divergent thermotolerance capacities, providing a strong rationale for evaluating multiple strains in stress-related studies (25). This intrinsic thermal tolerance has been associated with its ability to upregulate antioxidant activity and secrete organic acids under heat stress. Its ecological dominance has been further confirmed by long-term warming experiments, where it exhibited sustained survival under elevated temperatures (23). By modifying root architecture, improving nutrient and water acquisition, and activating stress-response systems, *C. geophilum* significantly enhances host resilience (6), underscoring its immense potential as a natural bolster against climate change. However, the precise transcriptional programming and enzymatic mechanisms by which it regulates proline and NO metabolism under heat stress remain unresolved. Elucidating these mechanisms represents a critical innovative step, promising to uncover novel physiological pathways and translate the fundamental symbiosis into targeted strategies for forest resilience.

To address the aforementioned questions, by employing *P. massoniana* as the host plant and six ecotypes of *C. geophilum* as experimental ECM fungi, this study incorporated two factorial combinations of temperature regimes (25°C and 35°C) and mycorrhizal inoculation treatments (control and *C. geophilum* inoculation). A comprehensive set of physiological and biochemical parameters was assessed, including plant growth performance, proline content, key substrates, and enzymatic activities related to proline metabolism, as well as endogenous NO levels and major NO synthase activities. More importantly, plant transcriptome sequencing (RNA-Seq) data were also obtained to elucidate the molecular underpinnings of the plant-microbe mutualism under temperature stress. Therefore, this study was designed to test three interconnected hypotheses that progress from physiological effects to underlying molecular mechanisms: (i) *C. geophilum* inoculation could enhance heat tolerance in *P. massoniana* by improving biomass, photosynthesis, and antioxidant capacity, while reducing oxidative damage as indicated by malondialdehyde (MDA) content; (ii) the enhanced thermotolerance could be facilitated by the symbiosis through its coordinated modulation of proline and nitric oxide (NO) metabolism; and (iii) the molecular basis of the *C. geophilum*-induced thermotolerance could be elucidated through key gene expression patterns responsible for the observed physiological and metabolic resilience. By validating these hypotheses, our findings aim to establish a mechanistic link between mycorrhizal symbiosis and host thermotolerance, thereby providing a robust scientific foundation for deploying *C. geophilum* as an ecological tool to enhance forest resilience under global warming.

## MATERIALS AND METHODS

### Inoculation of *P. massoniana* seedlings with ECM fungi

The strains of *C. geophilum* (C1–C6) were provided by the International Joint Research Laboratory of Symbiotic Organisms in Forestry at Fujian Agriculture and Forestry University, China. The strains were pre-cultivated on Modified Melin-Norkrans medium at 25°C for 45 days. The seeds of *P. massoniana* were sourced from Wuyi Mountain Farm in Zhangping City, Fujian Province, China, and the seedling cultivation methods were consistent with previous studies (26). After 30 days of cultivation, the seedlings were transplanted into culture boxes (23.5 × 8.5 × 1.6 cm, three seedlings per box), with a growth substrate consisting of autoclaved forest soil and Akadama soil (weight ratio 1:2), where the forest soil was collected from the forest land of Fujian Agriculture and Forestry University (0–20 cm depth). Fifteen days after transplantation, mycelial plugs (1 cm in diameter and 0.5 cm in thickness) were excised from the 45-day-old *C. geophilum* cultures of six different strains (C1–C6) using a sterile cork borer. For each of the six independent inoculation treatments, these plugs were applied directly onto the well-developed lateral root tips of three individual *P. massoniana* seedlings, covering a 1 × 1 cm area per root tip. The total inoculum quantity applied to each seedling was standardized to the mycelial biomass harvested from one full 9 cm diameter Petri dish (approximately 63.62 cm^2^ per seedling). Each of the six strains was applied to three replicate pots (three seedlings per pot), and since the primary objective was to compare inoculated vs non-inoculated seedlings under different temperature regimes, data from the six individual strain treatments were pooled into a single unified inoculation group for statistical analysis, yielding 54 seedlings in total (6 strains × 3 replicate pots × 3 seedlings per pot). The non-inoculated control (CK) was established by applying an equal amount of sterilized MMN medium to seedlings under the same conditions, with three replicate pots of three seedlings per pot. Combined with two temperature regimes (25°C and 35°C), the full experiment comprised 14 treatment combinations in total. After inoculation, the seedlings were cultivated in a greenhouse with environmental parameters of 25°C/23°C (day/night), light intensity of 300 μmol m^−2^ s^−1^, relative humidity of 70%, and a photoperiod of 16 h light/8 h dark. During the cultivation process, all plants were watered weekly with 200 mL of sterile water and once a month with 200 mL of Hoagland’s nutrient solution (volume ratio 1:1,000). The ectomycorrhizal colonization rate of *P. massoniana* seedlings was assessed under a stereomicroscope (OLYMPUS-S261, Olympus, Japan) at 10× magnification. For each seedling, approximately 300 root tips were randomly selected from the entire root system, and each tip was scored as either mycorrhizal or non-mycorrhizal based on characteristic ECM morphology (e.g., hyphal mantle formation and root tip swelling). The colonization rate was calculated as the number of mycorrhizal root tips divided by the total number of root tips examined, expressed as a percentage. Only seedlings with a colonization rate exceeding 80% were selected for subsequent experiments.

### Experiment setup of inoculation and temperature treatments

To determine the effects of *C. geophilum* on the growth, resistance indices, and proline metabolism of *P. massoniana* under high-temperature stress, we established inoculation and temperature treatments. The inoculation treatment included *P. massoniana* inoculated with six different ecotypes of *C. geophilum* strains (Inoculation C1–C6) and a non-inoculated control. The temperature treatment included two levels: a high-temperature condition (35°C) of 28 ± 1.0°C (15:00–9:00) and 35 ± 1.0°C (9:00–15:00), and a normal-temperature condition (25°C) of 20 ± 1.0°C (15:00–9:00) and 25 ± 1.0°C (9:00–15:00). The growth chambers for both temperature treatments had a photoperiod of 16 h and a photosynthetic photon flux density of 300 μmol m^−2^ s^−1^. The experiment consisted of 14 treatments, resulting from seven inoculation conditions (one control and six fungal strains) and two temperatures. Each treatment was replicated three times, with three *P. massoniana* seedlings per pot. After the completion of the inoculation with control and six strains, the seedlings were placed in incubators set for normal or high temperatures for a 60-day temperature treatment.

To minimize the influence of positional variation in light intensity and temperature distribution within each growth chamber, the positions of all pots within the same temperature treatment group were randomly rearranged on a weekly basis throughout the experimental period. The plants were irrigated with tap water every 10 days and with Hoagland’s nutrient solution every 30 days. After 2 months of temperature treatment, the roots were carefully washed with tap water to remove attached soil particles, followed by a thorough rinse with deionized water. All harvested plant samples were divided into two parts: one part for the determination of biomass indicators, and the other part immediately frozen in liquid nitrogen and stored at −80°C for the determination of physiological and biochemical indices.

### Measurements of plant biomass, photosynthetic parameters, malondialdehyde (MDA), and antioxidant enzyme activity

For each treatment, plants were sampled with their fresh weight immediately determined using an electronic balance after removing the attached soil particles from the roots. The seedlings were then placed in an oven, blanched at 105°C for 15 min, and dried at 80°C to a constant weight, with their dry weight measured.

Gas exchange parameters of the seedling needles of *P. massoniana* were measured using a portable Li-6400XT photosynthesis system (Li-COR, Lincoln, NE, USA). Photosynthetic parameters, including net photosynthesis rate (Pn), transpiration rate (Tr), stomatal conductance (Gs), and intercellular CO_2_ concentration (Ci), were determined for three *P. massoniana* seedlings per treatment between 09:00 and 12:00 using fresh current-year needles from the same seedlings subsequently used for biomass determination and physiological assays of MDA content and antioxidant enzyme activities. For each treatment (three replicate pots, three seedlings per pot), one healthy current-year needle was selected from each seedling, yielding nine needles per treatment. Gas exchange measurements were conducted on living plants immediately prior to harvest, after which the same seedlings were used for biomass determination and physiological assays of MDA content and antioxidant enzyme activities. Needles were excised from living plants and placed into the leaf chamber within 2 min of excision to minimize any effect on stomatal aperture. MDA content was determined using the thiobarbituric acid (TBA) method, which quantifies MDA based on its reaction with TBA to form a red complex with characteristic absorption at 532 nm (26, 27). Additionally, catalase (CAT) activity was measured using the ultraviolet absorption method, based on the principle that CAT decomposes H_2_O_2_, which has a characteristic absorption at 240 nm (28, 29). Peroxidase (POD) activity was determined using the guaiacol method, in which POD catalyzes the H_2_O_2_-dependent oxidation of guaiacol to produce a brown-colored product measurable at 470 nm (26, 29). Superoxide dismutase (SOD) activity was measured using the nitro blue tetrazolium (NBT) photochemical reduction method, based on the principle that SOD inhibits the light-driven reduction of NBT by a riboflavin-methionine system, with the degree of inhibition reflecting SOD activity (26, 30).

### Measurements of proline, NO, metabolic substrates, and NO-producing enzyme activities

A 0.1 g sample of homogenized tissue was weighed, with the Pro content in the shoot and root parts under different treatments determined using the modified ninhydrin colorimetric method with sulfosalicylic acid (28). The glutamate (Glu) content was determined using the modified ninhydrin colorimetric method in 0.2 g samples mixed with 2 mL of 0.05 mol/L PBS buffer at pH 7.8. The mixture was rapidly ground in an ice bath and then centrifuged at 4°C (8,000 × *g* for 10 min). The resulting supernatant was used as the extract. A 1 mL aliquot of the supernatant was placed in a test tube, with 200 μL of ninhydrin solution added, followed by heating in a 90°C water bath for 20 min. After cooling, the absorbance was measured at 569 nm.

The Orn content was determined using the ninhydrin colorimetric method adapted from Chinard (31), in which samples were homogenized in 10% acetic acid, evaporated to a final volume of 10 mL, and centrifuged at 4,000 rpm for 10 min at 4°C; color development was performed using a ninhydrin solution prepared in H_3_PO_4_-acetone (1:3) at 100°C for 1 h, with absorbance measured at 510 nm (31). The activity of Δ1-pyrroline-5-carboxylate synthetase (P5CS) was determined using the hydroxylamine colorimetric method adapted from Hayzer and Leisinger (32), with modifications including the addition of 2% PVP to the extraction buffer (0.5 mol/L Tris-HCl, pH 7.5, 10 mmol/L MgCl_2_, and 2 mmol/L β-mercaptoethanol) and the use of a FeCl_3_-trichloroacetic acid stopping solution, with absorbance measured at 535 nm (32). The activity of ornithine aminotransferase (OAT) was measured following Hervieu et al. (33) with slight modifications, including the addition of 1 mmol/L β-mercaptoethanol to the extraction buffer and dissolution of the reaction precipitate in 1.5 mL absolute ethanol prior to absorbance measurement at 510 nm (33). Proline dehydrogenase (ProDH) activity was determined according to Lea et al. (34) with modifications, using a Na_2_CO_3_-NaHCO_3_ buffer (pH 10.3) as the reaction system with 0.5% Triton X-100 added to the extraction buffer for membrane protein solubilization, and absorbance measured at 600 nm (34). The content of nitric oxide (NO) was measured using an NO content assay kit (A012-1-2) from Nanjing Jiancheng Bioengineering Institute. The activity of nitrate reductase (NR) in the shoot and root parts under different treatments was determined using the sulfanilamide colorimetric method (35). The activity of nitric oxide synthase (NOS) was measured according to published methods (35).

### Plant root transcriptome sequencing analysis

Total RNA was isolated from the roots of *P. massoniana* seedlings utilizing the CLB-Adlai RN40 reagent system (RN40, Adlai). The concentration and purity of the extracted RNA were determined using a NanoDrop 2000 spectrophotometer (Thermo Fisher Scientific, Waltham, MA, USA), while the Bioanalyzer 2100 (Agilent Technologies, Santa Clara, CA, USA) was employed to assess RNA integrity. Biomarker Technologies (Beijing, China) carried out the construction of the complementary DNA (cDNA) library, subsequent quality checks, and sequencing on an Illumina NovaSeq 6000 platform, adhering to established protocols. Raw sequencing reads were filtered by BMKCloud (Beijing, China) following standard quality control procedures. The filtration criteria were as follows: (i) removal of reads containing adapter sequences; (ii) removal of reads with more than 10% unknown bases (N); and (iii) removal of low-quality reads in which more than 50% of bases had a Phred quality score ≤10 (Q10). Only clean reads passing all filtration criteria were retained for subsequent analysis. Clean reads were *de novo* assembled into a unigene library using Trinity with the following key parameters: --min_contig_length 200 (minimum contig length of 200 bp) and --group_pairs_distance 500, with all remaining parameters set to default values (7, 25). Transcript abundance was estimated by aligning clean reads back to the Trinity assembly using Bowtie2, followed by quantification with RSEM using default parameters.

Differential expression analysis was performed using DESeq2 (v1.44.0), which normalized raw read counts and calculated log_2_ fold changes (log_2_FC) and adjusted significance levels (FDR) based on the Wald test with the Benjamini–Hochberg correction. Genes with |log_2_FC| ≥ 1 and FDR < 0.05 were considered significantly differentially expressed. For the volcano plot, log_2_FC and −log_10_(FDR) were used as the *x*- and *y*-axes, respectively, with threshold reference lines added; significantly up- and downregulated genes were highlighted in distinct colors, while non-significant genes were shown in gray. Enrichment analyses of GO and KEGG pathways were conducted on the identified DEGs using clusterProfiler with hypergeometric testing, followed by FDR correction. Enriched terms were ranked by gene ratio or FDR, and significant categories were visualized using bubble plots, where the x-axis represented gene ratio, the *y*-axis displayed pathway names, bubble size indicated the number of enriched genes, and bubble color encoded −log_10_(FDR). All visualizations were generated in R (v4.3.3) using ggplot2 and the dotplot() function in clusterProfiler (v4.12.0), with consistent parameter settings to ensure reproducibility.

### Statistical analysis

Statistical analysis was performed with SPSS software version 26.0, employing one-way analysis of variance (ANOVA) followed by Tukey’s honestly significant difference (HSD) test for multiple comparison tests.

## RESULTS

### Effects of inoculation and heat stress on seedling morphology, biomass, and photosynthesis

**Fig 1:**
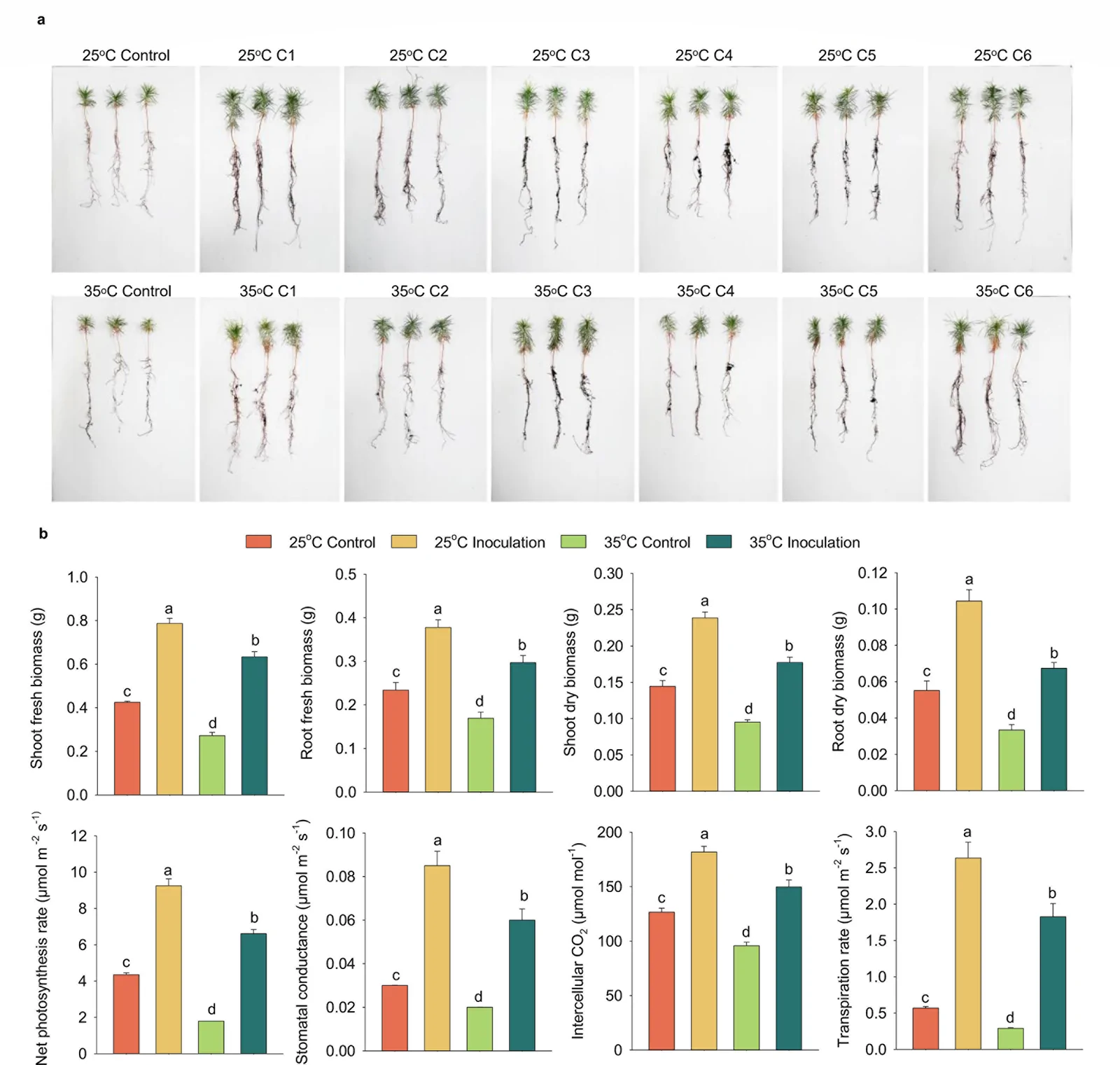
Effects of *Cenococcum geophilum* inoculation and temperature on seedling morphology and growth of *Pinus massoniana*. (**a**) Morphology of seedlings inoculated with six ecotypes of *Cenococcum geophilum* and non-inoculated controls under 25°C and 35°C conditions. (**b**) Biomass and gas-exchange traits, including net photosynthetic rate (Pn, μmol CO_2_ m^−2^ s^−1^), stomatal conductance (Gs, mol H_2_O m^−2^ s^−1^), intercellular CO_2_ concentration (Ci, μmol CO_2_ mol^−1^), and transpiration rate (Tr, mmol H_2_O m^−2^ s^−1^), across treatments. Different letters indicate significant differences at *P* < 0.05.

The inoculation of *C. geophilum* and higher temperature treatment showed significant impacts on the morphology of *P. massoniana* seedlings (Fig. 1a). Compared to *P. massoniana* seedlings at 25°C (25°C Control), *C. geophilum*-inoculated seedlings (25°C C1–C6) exhibited enhanced growth, including greater shoot height, denser foliage, and increased root length and branching. Under high-temperature stress, seedlings (35°C control) exhibited significant growth inhibition, characterized by needle curling, progressive chlorosis from basal to apical leaves, partial scorch, leaf abscission, and reduced root development. In contrast, inoculated seedlings (35°C C1–C6) maintained superior needle morphology and greenness, with reduced curling, chlorosis, and defoliation, alongside improved root length and branching, indicating that *C. geophilum* inoculation alleviates high-temperature stress in *P. massoniana* seedlings.

Additionally, seedling fresh/dry biomass of shoot and root along with four photosynthetic parameters, including net photosynthetic rate, stomatal conductance, intercellular CO_2_ concentration, and transpiration rate, were significantly different among inoculation treatments and temperature regimes (Fig. 1b, *P* < 0.05). Specifically, compared to 25°C control, inoculated seedlings (25°C Inoculation) exhibited greater fresh/dry biomass of shoot and root by 63% and 87%, respectively, as well as enhanced net photosynthetic rate (Pn) by 113%, stomatal conductance (Gs) by 183%, intercellular CO_2_ concentration (Ci) by 43%, and transpiration rate (Tr) by 360% (*P* < 0.05).

Exposure to high temperature significantly suppressed biomass accumulation and photosynthetic capacity of seedlings (35°C control), with declines across all measured parameters by 24–59% (*P* < 0.05). However, inoculation with *C. geophilum* markedly alleviated these heat-induced reductions. Compared with 35°C control, inoculated seedlings (35°C Inoculation) achieved increases in fresh/dry biomass of shoot and root by 80% and 118%, respectively, as well as significantly a half- to fivefold enhancement in tested photosynthetic parameters (*P* < 0.05). These results suggest that *C. geophilum* inoculation substantially mitigates, though it cannot completely prevent, the detrimental effects of higher temperature on the biomass and photosynthesis of *P. massoniana* seedlings.

### Effects of inoculation and heat stress on seedling oxidative stress, enzyme activities, and NO metabolisms

**Fig 2:**
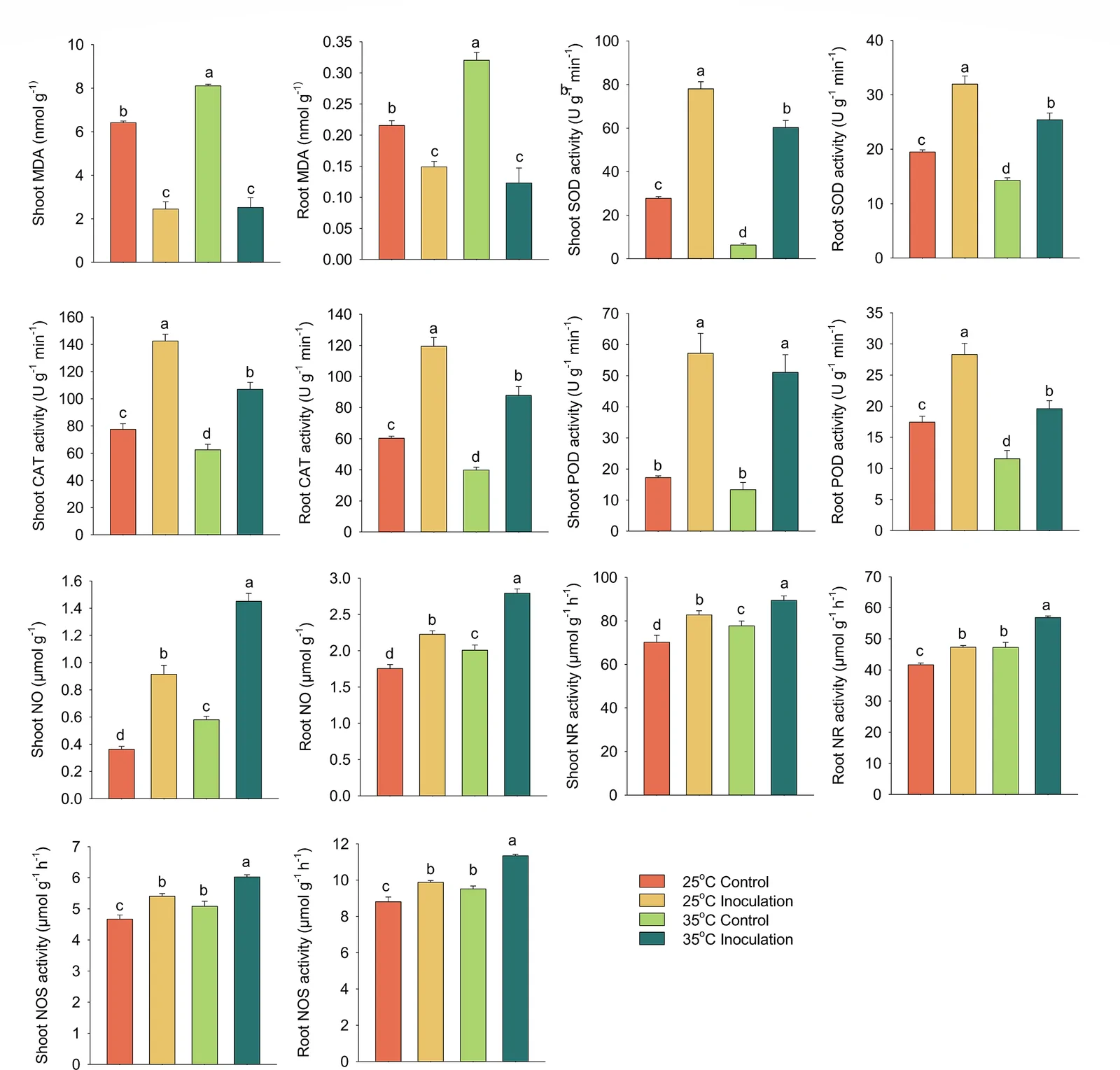
Malondialdehyde (MDA), activities of antioxidant enzymes of SOD, CAT, POD, and NO-related indices of NO, nitrate reductase (NR) and nitric oxide synthase (NOS) activities in shoots and roots of *Pinus massoniana* seedlings inoculated with *Cenococcum geophilum* and non-inoculated controls under 25°C and 35°C conditions. Letters indicate significant differences at *P* < 0.05.

Compared with 25°C control, 25°C inoculation substantially led to a reduction of shoot and root MDA levels by 62% and 31%, respectively, while rose antioxidant enzyme activities of SOD by 64–181%, CAT by 84–98%, and POD by 62–232% (Fig. 2, *P* < 0.05). High-temperature stress significantly elevated both shoot and root MDA by 26–49% but decreased antioxidant enzyme activities of SOD, CAT, and POD in both tissues by 27–77%, 19–34%, and 22–34%, respectively (*P* < 0.05). However, 35°C inoculation consistently counteracted these negative effects of heat stress, with enhanced antioxidant enzyme activities of SOD, CAT, and POD in seedling shoots and roots by 71–862% and 70–121%, respectively, compared with 35°C control (*P* < 0.05).

In addition, 25°C inoculation led to pronounced increases in NO accumulation by overall 27–152%, NR and NOS activities by 13–18% and 12–16%, respectively, in seedling shoots and roots than 25°C control (*P* < 0.05). High-temperature stress also stimulated seedling NO accumulation by 14–60%, while NR activity and NOS activity exhibited modest rises of 10–13% and 8–9%, respectively, compared with 25°C control (*P* < 0.05). More importantly, 35°C inoculation further resulted in 39–150% elevation in both shoot and root NO accumulation, as well as increases in seedling NR activity by 15–20% and NOS activity by 18–19% than 35°C control (*P* < 0.05).

### Effects of inoculation and heat stress on seedling proline, substrates, and enzyme activities

**Fig 3:**
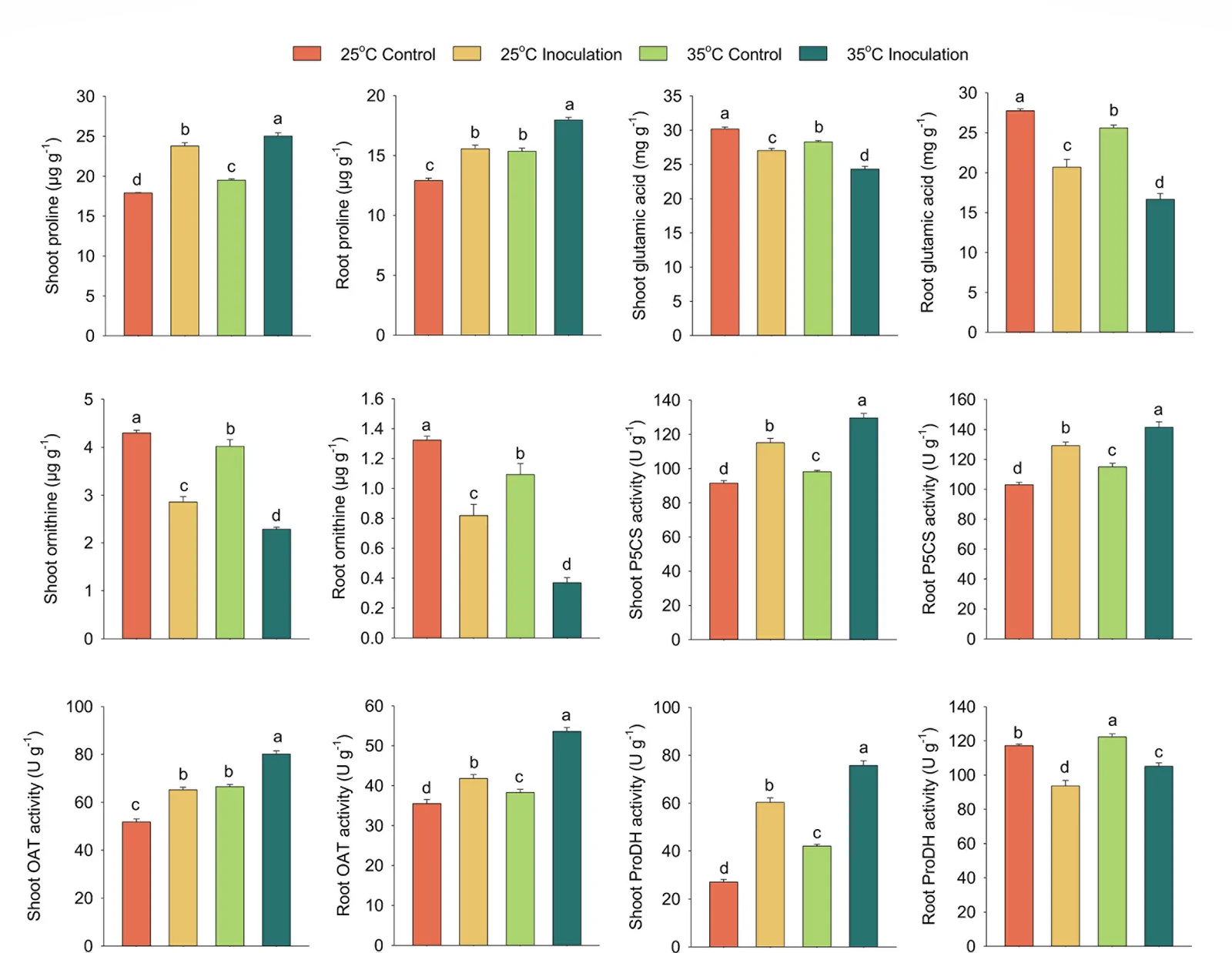
Proline, glutamic acid, ornithine, and activities of related metabolic enzymes of Δ1-pyrroline-5-carboxylate synthetase (P5CS), ornithine aminotransferase (OAT), proline dehydrogenase (ProDH) in shoots and roots of *Pinus massoniana* seedlings inoculated with *Cenococcum geophilum* and non-inoculated controls under 25°C and 35°C conditions. Letters indicate significant differences at *P* < 0.05.

At 25°C, inoculation markedly promoted proline (Pro) accumulation in the seedlings, with increases of shoot Pro from 18 to 24 µg g^−1^ by 33%, and root Pro from 13 to 16 µg g^−1^ by 21% (Fig. 3, *P* < 0.05). Compared with 25°C inoculation, 35°C inoculation further stimulated Pro accumulation in seedling shoots and roots by 5% and 15%, respectively. By contrast, 25°C inoculation led to significant declines of seedling shoot and root glutamic acid (Glu) and ornithine (Orn) levels, the substrates to produce Pro, by 10–25% and 33–38%, respectively, which were further restrained sharply by 14–35% and 43–66% in seedling shoots and roots, respectively, under 35°C inoculation (*P* < 0.05).

Inoculation at 25°C also significantly increased P5CS and OAT activities, two important enzymes related to Pro production, by 17–26% in both seedling shoots and roots (*P* < 0.05). Even under heat stress, 35°C inoculation further enhanced the enzyme activity of P5CS and OAT by 23–32% and 20–40%, respectively. Interestingly, compared with 25°C control, inoculation at both 25°C and 35°C significantly increased ProDH activity, the key enzyme to degrade Pro, by 123% and 80%, respectively, only in seedling shoots, but decreased it by 20% and 14%, respectively, in seedling roots (*P* < 0.05).

### Effects of inoculation on seedling transcriptomes at 25°C and 35°C

**Fig 4:**
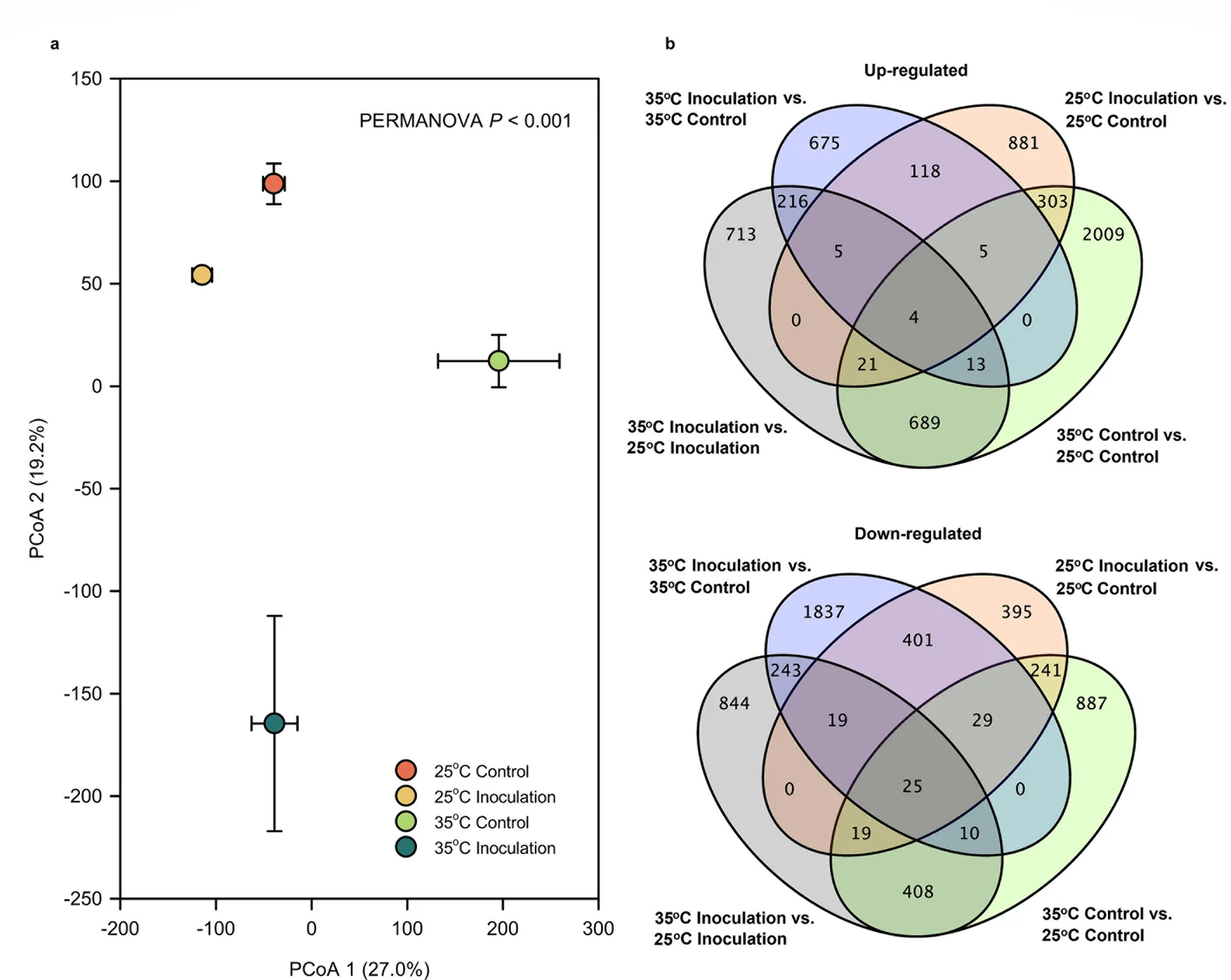
Effects of *Cenococcum geophilum* inoculation and temperature on the profiles of differentially expressed genes (DEGs) of transcriptomes in *Pinus massoniana* roots. (**a**) Principal coordinates analysis (PCoA) showing significant variations in DEG profiles of *Pinus massoniana* seedlings inoculated with *Cenococcum geophilum* and non-inoculated controls under 25°C and 35°C conditions. PERMANOVA *P* < 0.001 was provided. (**b**) Venn diagrams displaying the number of upregulated (top panel) and downregulated (bottom panel) DEGs in pairwise comparisons among the four treatments of 25°C Control, 25°C Inoculation, 35°C Control, and 35°C Inoculation.

**Fig 5:**
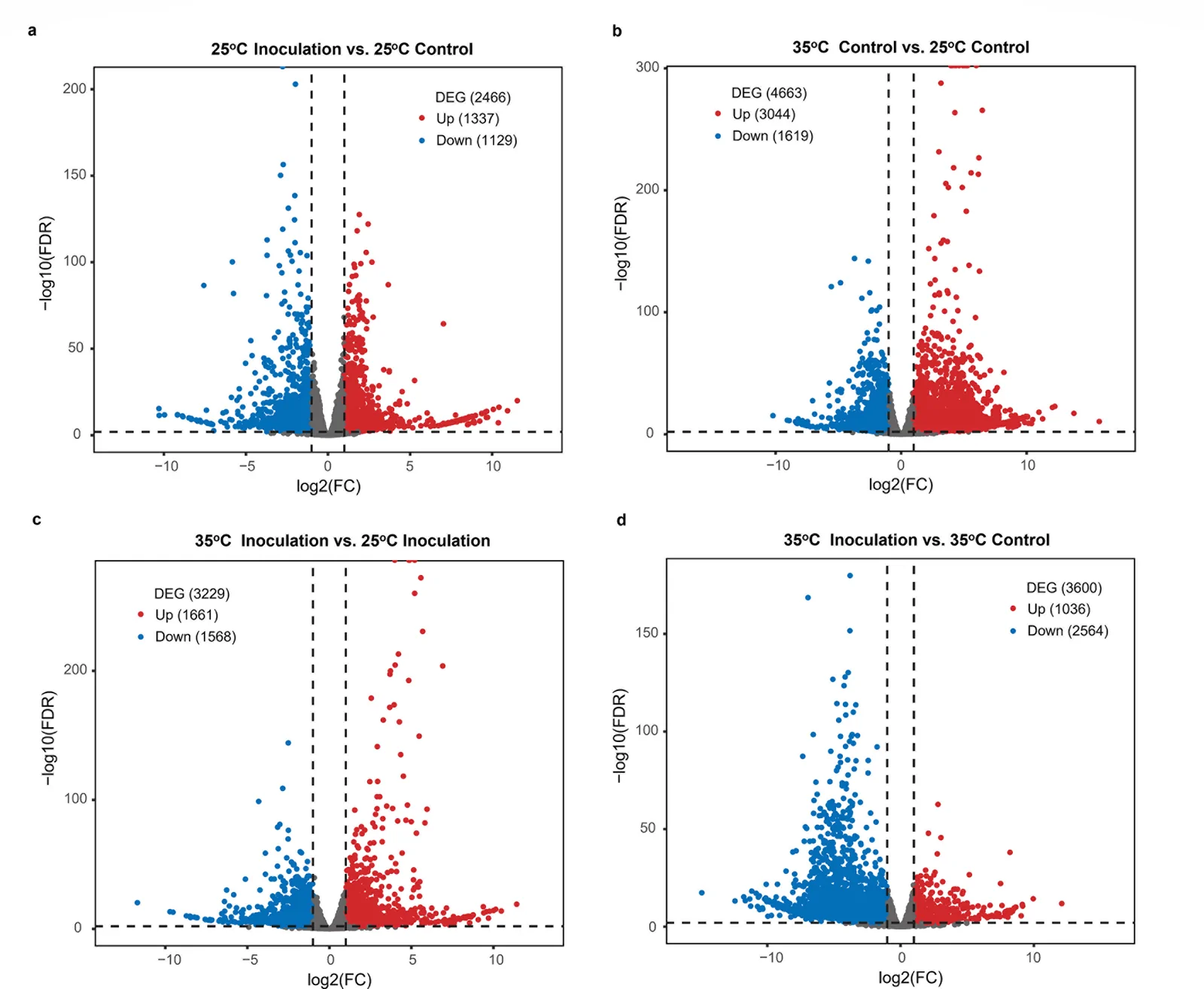
Differentially expressed genes (DEGs) of transcriptomes in *Pinus massoniana* roots under *Cenococcum geophilum* inoculation and temperature treatments. (**a–d**) Volcano plots showing pairwise comparisons of DEG profiles among the four treatments of 25°C Control, 25°C Inoculation, 35°C Control, and 35°C Inoculation. In each plot, the *x*-axis represents log_2_ fold-change (log_2_FC) and the *y*-axis represents –log_10_(FDR), where FDR denotes the false discovery rate. Dashed lines indicate the significance thresholds (|log_2_FC| ≥ 1 and FDR < 0.05). Significantly upregulated and downregulated DEGs are highlighted in red and blue, respectively, while non-significant genes are shown in gray.

Principal coordinate analysis (PCoA) revealed significant variations in the profiles of differentially expressed genes (DEGs) affected by inoculation and heat stress (Fig. 4a). Specifically, 25°C control and 25°C inoculation clustered relatively closer, while 35°C control and 35°C inoculation were more distantly distributed, showing more significant effects of higher temperature than inoculation treatments (PERMANOVA *P* < 0.001). The Venn diagrams (Fig. 4b) and Volcano plots (Fig. 5) revealed the overlaps and numbers of up-/downregulation DEGs in response to inoculation at 25°C and 35°C. The complete lists of pathways of differentially expressed genes (DEGs) for all comparisons are provided in Table S1.

**Fig 6:**
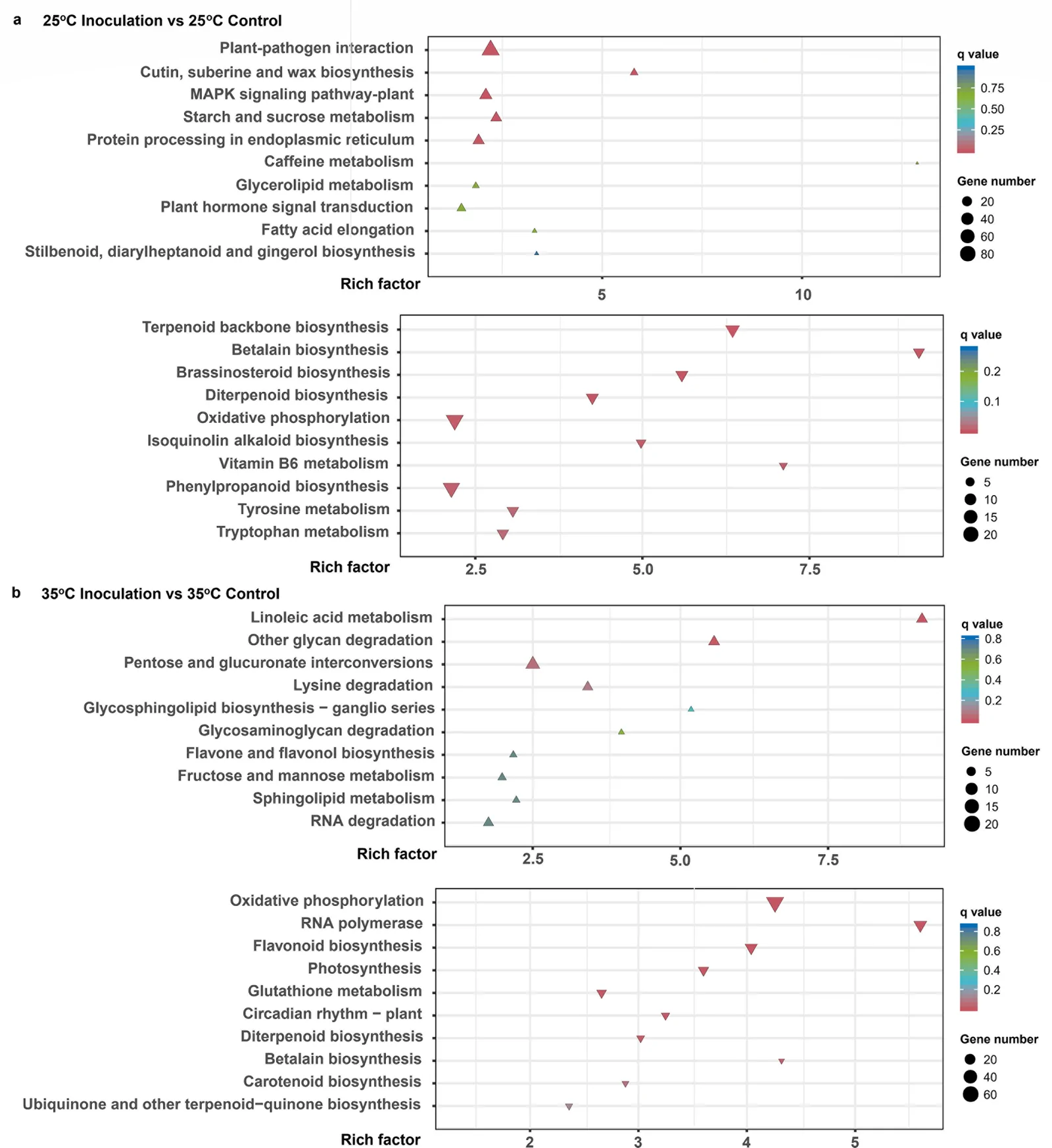
The enrichment analysis of differentially expressed genes (DEGs) of transcriptomes in *Pinus massoniana* roots under *Cenococcum geophilum* inoculation and temperature treatments. Two panels of upregulated (top) and downregulated (bottom) showing KEGG pathways of DEGs significantly enriched by the 25°C Inoculation vs 25°C Control comparison (**a**) and the 35°C Inoculation vs 35°C Control comparison (**b**). In each plot, the symbol size represents the number of DEGs enriched in the pathway annotated in KEGG, with symbol colors representing the corrected *P* value (*q* value) of the enrichment and Rich factors referring to the ratio of DEG count enriched in the pathway to the total gene count of that pathway.

At 25°C, inoculation triggered 1337 upregulation genes and 1129 downregulated genes, of which 881 and 395 genes of up- and downregulation, respectively, were uniquely found in seedlings affected by inoculation (25°C Control vs 25°C Inoculation). These upregulation genes were mainly related to the plant functions in plant-pathogen interaction (118 genes), cutin, suberin and wax biosynthesis (18 genes), MAPK signaling pathway-plant (60 genes), starch and sucrose metabolism (36 genes), and protein processing in endoplasmic reticulum (51 genes) (Fig. 6a). Downregulation genes were associated with biosynthesis pathways of terpenoid backbone (16 genes), betalain (9 genes), brassinosteroid (12 genes), diterpenoid (12 genes), and isoquinoline alkaloid (8 genes), as well as the oxidative phosphorylation (26 genes). Notably, key candidate genes were upregulated, including multiple heat shock proteins (e.g., HSP70 and HSP90 family members) and transcription factors such as WRKY (e.g., *WRKY19* and *WRKY30*), ERF (e.g., *ERF016* and *ERF017*), MYB, and NAC family members, which likely contribute to the priming of antioxidant defenses and defense signaling observed physiologically under normal conditions. More key candidate genes for selected pathways are listed in Table S1.

At 35°C, inoculation triggered 1,036 upregulation genes and 2,564 downregulated genes, of which 675 and 1,837 genes of up- and downregulation, respectively, were uniquely found in seedlings responding to inoculation (35°C Control vs 35°C Inoculation). These upregulation genes were mainly related to the plant functions of linoleic acid metabolism (10 genes), pentose and glucuronate interconversions (15 genes), degradation of lysine (9 genes), glycosaminoglycan degradation (3 genes), and other glycan (1 gene) (Fig. 6b). Downregulation genes were associated with oxidative phosphorylation (64 genes), RNA polymerase (33 genes), flavonoid biosynthesis (35 genes), photosynthesis (21 genes), glutathione metabolism (17 genes), and plant circadian rhythm (15 genes). Among them, several peroxidase genes and transcription factors (e.g., *WRKY51*, ERF family, *MYB61*, and *NAC007*) were prominently induced, as well as antioxidant-related peroxidases (e.g., *PER19* and *PER9*), reinforcing the observed enhancements in antioxidant enzyme activities (SOD, CAT, and POD) and NO signaling under heat stress.

### Effects of heat stress on seedling transcriptomes with/without inoculation

**Fig 7:**
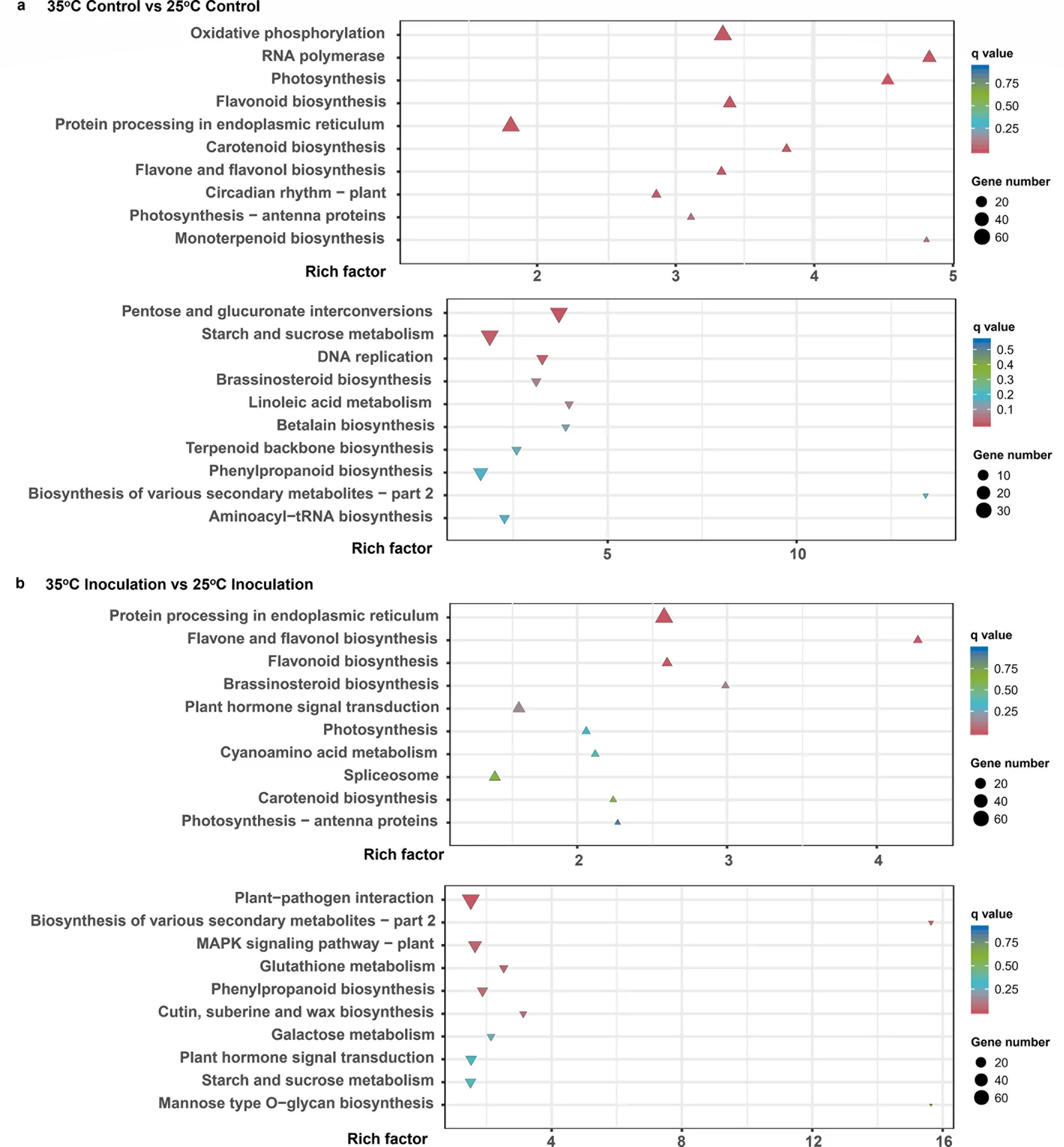
The enrichment analysis of differentially expressed genes (DEGs) of transcriptomes in *Pinus massoniana* roots under *Cenococcum geophilum* inoculation and temperature treatments. Two panels of upregulated (top) and downregulated (bottom) showing KEGG pathways of DEGs significantly enriched by the 35°C Control vs 25°C Control comparison (**a**) and the 35°C Inoculation vs 25°C Inoculation comparison (**b**). In each plot, the symbol size represents the number of DEGs enriched in the pathway annotated in KEGG, with symbol colors representing the corrected *P* value (*q* value) of the enrichment and Rich factors referring to the ratio of DEG count enriched in the pathway to the total gene count of that pathway.

In the control, higher temperature resulted in 3,044 upregulation genes and 1,619 downregulated genes (Fig. 5b), of which 2,009 and 887 genes of up- and downregulation, respectively (Fig. 4b), were uniquely found in seedlings affected by heat stress without inoculation (25°C Control vs 35°C Control). These upregulation genes were mainly related to the oxidative phosphorylation (64 genes), RNA polymerase (35 genes), photosynthesis (31 genes), protein processing in endoplasmic reticulum (72 genes), and biosynthesis of flavonoid (35 genes) and carotenoid (16 genes) were also upregulated (Fig. 7a). Downregulation genes were associated with pentose and glucuronate interconversions (15 genes), starch and sucrose metabolism (41 genes), DNA replication (12 genes), brassinosteroid biosynthesis (11 genes), and linoleic acid metabolism (10 genes). Strong induction of heat shock protein genes (e.g., multiple small HSPs like *HSP17.3*, *HSP17.7*, and HSP70 family) and transcription factors (WRKY, ERF, MYB, and NAC) was evident, consistent with the activation of stress-responsive pathways (Table S1).

When seedlings were inoculated with fungi, exposure to heat stress led to 1,661 upregulation genes and 1,568 downregulated genes (Fig. 5c), of which 675 and 1,837 genes of up- and downregulation (Fig. 4b), respectively, were uniquely found in seedlings responding to inoculation and higher temperature together (25°C Inoculation vs 35°C Inoculation). These upregulation genes were mainly related to the plant functions of protein processing in endoplasmic reticulum (72 genes), biosynthesis of carotenoid (16 genes), flavone and flavonol (12 genes), flavonoid (18 genes), brassinosteroid (8 genes), plant hormone signal transduction (29 genes), and photosynthesis (4 genes) (Fig. 7a). Downregulation genes were associated with plant-pathogen interaction (32 genes), plant MAPK signaling pathway (23 genes), glutathione metabolism (8 genes), biosynthesis of phenylpropanoid (16 genes), and cutin, suberin, and wax (5 genes).

**Fig 8:**
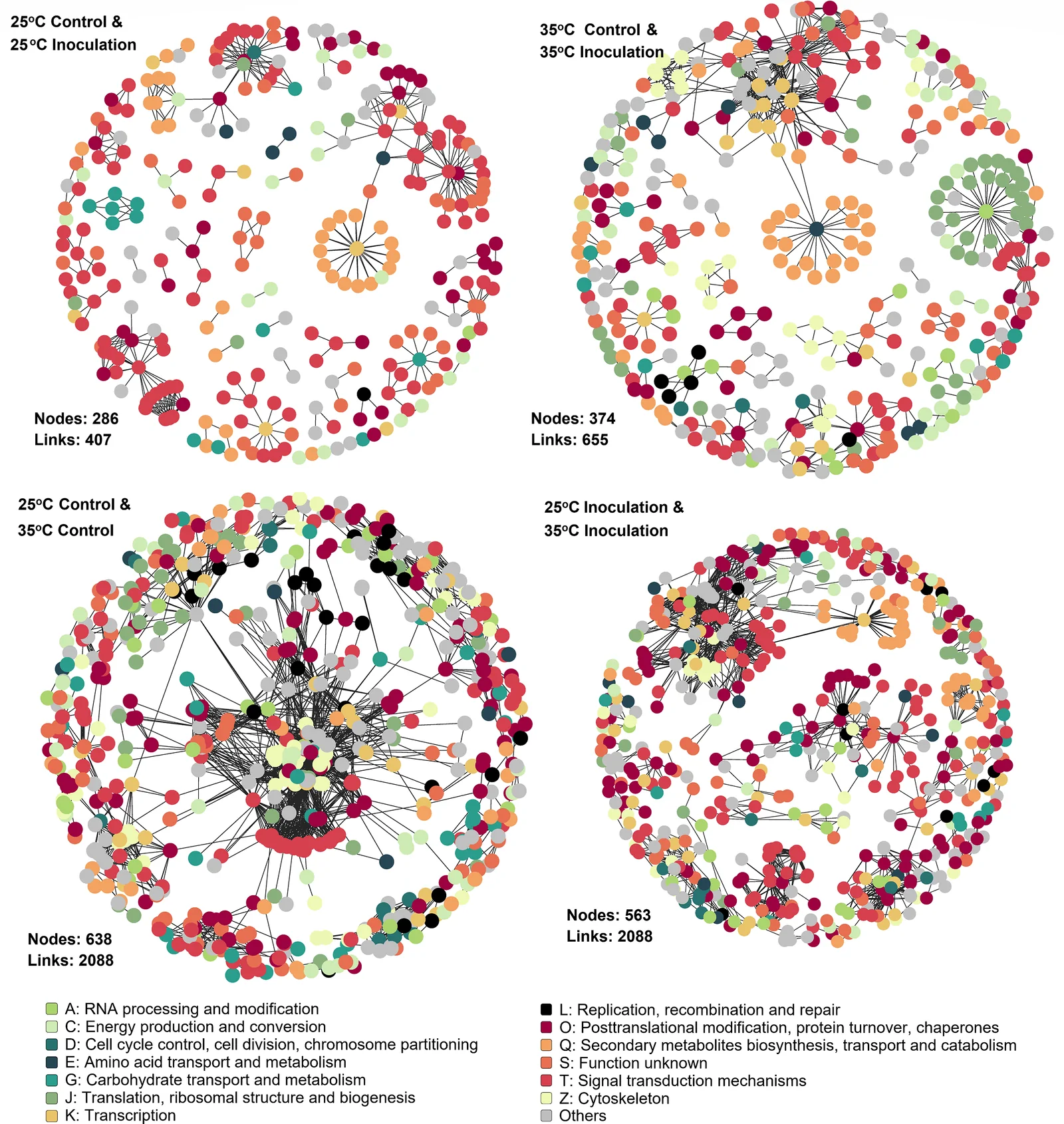
Gene co-expression networks of differentially expressed genes (DEGs) in *Pinus massoniana* roots under *Cenococcum geophilum* inoculation and temperature treatments. The four panels correspond to the co-expression networks of DEGs from four specific treatment combinations of 25°C Control and 25°C Inoculation (top-left), 35°C Control and 35°C Inoculation (top-right), 25°C Control and 35°C Control (bottom-left), and 25°C Inoculation and 35°C Inoculation (bottom-right). In each network, nodes represent individual DEGs, and links indicate significant co-expression correlations between genes. Node colors correspond to different classified functional groups. The total number of nodes and links involved in each network is displayed in the bottom-left corner of the respective panel.

Significant differences were observed in the functional networks of DEGs under different treatments (Fig. 8). In the 25°C Control vs Inoculated groups, the network was relatively small (286 nodes, 407 links) and dispersed, mainly associated with signal transduction mechanisms and the biosynthesis, transport, and catabolism of secondary metabolites. Under 35°C, network complexity increased (374 nodes, 655 links), with stronger connections among RNA processing, cytoskeleton organization, and signal transduction.

When comparing temperature effects, the 25°C vs 35°C control groups exhibited the most complex network (638 nodes, 2,088 links), characterized by dense interconnections particularly related to replication, recombination and repair, as well as posttranslational modification, protein turnover, and chaperone functions. In the 25°C vs 35°C inoculated groups, the extensive network (563 nodes, 2,088 links) exhibited a more modular organization, with enrichment mainly in carbohydrate transport and metabolism, and secondary metabolite biosynthesis, transport, and catabolism.

## DISCUSSION

### Effects of heat stress on plants’ physiological and biochemical parameters

Overall, transferring *P. massoniana* seedlings from 25°C to 35°C resulted in broad and consistent deterioration across multiple physiological dimensions. Shoot and root dry biomass declined by approximately 26% and 35%, respectively, accompanied by marked reductions in net photosynthetic rate, stomatal conductance, intercellular CO_2_ concentration, and transpiration rate. Concurrently, MDA content increased significantly in both tissues, while the activities of key antioxidant enzymes (SOD, CAT, and POD) paradoxically decreased, indicating that prolonged heat exposure impaired rather than stimulated the plant’s oxidative defense capacity. In contrast, ECM inoculation consistently buffered against these heat-induced deteriorations under both temperature regimes, restoring antioxidant enzyme activities, reducing oxidative damage, and supporting biomass accumulation and photosynthetic function. This overarching pattern establishes that *C. geophilum* inoculation acts as a systemic buffer against the detrimental physiological consequences of heat stress in *P. massoniana*, providing the broader context within which the specific mechanistic details discussed below should be interpreted. Our results showed that high temperature stress had significant impacts on plant growth and photosynthetic physiology (Fig. 1), mainly due to reduced photosynthesis and disrupted water and energy balance (36–38). Consistent with reports that heat stress disproportionately affects roots (39, 40), we also found that root dry weight decreased more markedly (35.43%) than shoot dry weight (25.73%), highlighting the vulnerability of the root system and its consequent limitation on overall plant performance.

Elevated temperature significantly impaired gas exchange in *P. massoniana*, reducing stomatal conductance (Gs) by 33.33% and intercellular CO₂ concentration (Ci) by 24.47% (Fig. 1). This indicates stomatal limitation as the primary constraint on photosynthesis under heat stress (41). Interestingly, despite the physiological suppression, genes related to photosynthesis-antenna proteins were upregulated (Fig. 7a), suggesting a transcriptional compensatory response to enhance light capture and electron transport under CO_2_ limitation (37, 41). However, this attempted recovery was ultimately ineffective, as the concurrent downregulation of starch, sucrose, and pentose metabolism pathways restricted carbon utilization (Fig. 7a), leading to energy imbalance and photosynthetic inefficiency.

High temperature induced severe oxidative stress in *P. massoniana*, as confirmed by elevated MDA levels (Fig. 2), a biomarker of membrane damage (42, 43). Paradoxically, the activities of key antioxidant enzymes (SOD, CAT, and POD) decreased significantly (Fig. 2), contrasting with the typical response reported in many species (44, 45). We speculate that prolonged heat stress may impair enzymatic function through protein inactivation or synthesis inhibition.

In plant responses to heat stress, NO plays a central role by modulating ROS levels, heat shock protein synthesis, and hormone signaling (20). In *P. massoniana*, warming increased NO levels, consistent with elevated NR and NOS activities (Fig. 2). In parallel, proline accumulation was tissue-specific, with roots showing higher levels (18.90%) than shoots (8.96%), linked to stronger P5CS activation in roots and greater OAT induction in shoots (Fig. 3). Notably, ProDH activity increased under warming (16), potentially to maintain redox homeostasis via proline degradation rather than solely for accumulation (46, 47). These results indicate that proline metabolism is spatially coordinated in *P. massoniana* to balance osmoprotection and redox adjustment during heat stress.

### Effects of inoculation on plants’ physiological and biochemical parameters

Our study shows that ECM inoculation not only promotes host growth but also enhances antioxidant defenses under thermal stress. The extraradical hyphal network of ECM fungi improves nutrient and water acquisition, directly supporting biomass accumulation (48–50). Complementing these nutritional benefits, ECM symbiosis significantly boosts the antioxidant capacity of hosts, as evidenced in poplar (51) and pine (52) studies showing that ECM colonization consistently elevates key enzyme activities (e.g., SOD, CAT, and POD) to mitigate oxidative damage. Consistent with these findings, our findings in *P. massoniana* revealed even more pronounced increases in SOD (64–181%), CAT (84–98%), and POD (62–232%) activities (Fig. 2), further corroborating the pivotal role of ECM-mediated antioxidant pathways in strengthening host resilience against environmental stresses.

Beyond the antioxidant enzyme system, our results indicate that ECM inoculation markedly alters NO metabolism in host plants. By enhancing host acquisition and transport of inorganic nitrogen, especially nitrate, ECM fungi increase substrate availability and activity of nitrate reductase (NR) in host roots, thereby indirectly elevating NO flux through the NR-dependent pathway (21, 53). In parallel, certain fungi are capable of producing NO themselves or its metabolic precursors, which may cross the symbiotic interface to influence host cellular NO levels or induce the expression of host NO biosynthetic enzymes (21, 54). Consequently, ECM symbiosis reshapes host NO dynamics at multiple levels, including nitrogen supply, NR activity, and fungal NO metabolism.

Alterations in NO levels further trigger downstream reprogramming of antioxidant defenses, constituting a key mechanism by which ECM enhances host stress tolerance. In *P. massoniana* under heat stress, ECM inoculation increased NO content by 27–152%, accompanied by 13–20% and 12–19% increases in NR and NOS activities, respectively (Fig. 2). These findings suggest that ECM symbiosis not only augments NO biosynthetic capacity but also potentially modulates the transcriptional regulation of antioxidant-related genes through NO signaling networks (55). This connection is reinforced by findings in drought-stressed poplar, where elevated NO coincided with higher SOD and CAT activities, confirming the coordinated upregulation of NO metabolism and antioxidant defenses in ECM symbiosis (56, 57). Collectively, these findings support a coherent mechanistic framework, showing that ECM symbiosis elevates host NO levels via improved nitrogen supply, which in turn activates antioxidant gene expression and enzyme activities, stabilizes ROS homeostasis, and ultimately enhances the host plant’s adaptive capacity under environmental stresses (21).

Integrating with the previously discussed NO and antioxidant pathways, ECM fungi further establish a coordinated regulatory network that optimizes proline metabolism for enhanced stress tolerance (10, 58, 59). Our research reveals that this regulation is not uniform but exhibits precise spatiotemporal control. In *P. massoniana*, ECM inoculation elicited opposing ProDH activity patterns, upregulation in shoots and downregulation in roots, highlighting a tissue-specific strategy (Fig. 3). This mirrors findings in ECM-colonized poplar and eucalyptus, where ProDH is activated in leaves to support energy metabolism via proline degradation, and simultaneously repressed in roots to bolster proline accumulation for osmotic adjustment and cellular protection (58–60). Collectively, these findings indicate that ECM symbiosis coordinates proline synthesis and degradation in a tissue-specific manner, thereby meeting the energetic demands of host plants while ensuring osmotic stability and enhanced stress tolerance.

### Effects of inoculation and heat stress on plants’ root transcriptomes

At 25°C, ECM inoculation also triggered a systemic transcriptomic reprogramming in *P. massoniana* roots, with upregulated genes predominantly enriched in defense and signaling pathways, epidermal protective structure formation, carbohydrate metabolism, and protein processing in the endoplasmic reticulum (Fig. 4 and 6). The first module involves activation of defense signaling, including upregulated plant-pathogen interactions (118 genes) and MAPK signaling (60 genes), indicating an enhanced defensive state of hosts (61). The second module includes the upregulation of 18 genes involved in cutin, suberin, and wax biosynthesis, thereby strengthening the root epidermis against water loss (62, 63). The third module encompasses metabolic optimization with upregulated starch and sucrose metabolism (36 genes) and protein processing (51 genes), ensuring efficient carbohydrate storage and utilization while enhancing protein folding and secretion efficiency (11). The early activation of MAPK signaling at 25°C may help establish a primed defensive state, which could partly underlie the higher baseline antioxidant enzyme activities (SOD, CAT, and POD) observed in ECM-inoculated seedlings even under normal temperature (Fig. 2), given that MAPK cascades are recognized as upstream regulators of antioxidant defense gene expression under stress conditions (64). Together, these transcriptional adjustments demonstrate that ECM symbiosis systemically primes host plants by simultaneously activating immune, structural, and metabolic pathways, even in the absence of immediate stress.

In parallel to the upregulation of defense and metabolic genes, ECM inoculation suppressed specific pathways in secondary metabolism and energy generation (Fig. 6). Downregulation was observed in terpenoid and alkaloid biosynthesis genes, indicating a shift in resource allocation from specialized metabolites toward growth and defense. Furthermore, the suppression of 26 oxidative phosphorylation genes likely reduces mitochondrial ROS production, complementing the activated antioxidant system to mitigate oxidative stress (65, 66). This suppression of oxidative phosphorylation genes may also partly contribute to the reduced MDA levels observed in ECM-inoculated seedlings at 25°C (Fig. 2), suggesting that mitochondrial ROS limitation operates as a basal protective mechanism even under non-stress conditions (24). This coordinated downregulation reflects a metabolic rebalancing that optimizes energy use and minimizes internal oxidative damage.

Under heat stress at 35°C, ECM inoculation induced substantial transcriptional reprogramming in the host seedlings (Fig. 4 and 7), toward pathways involved in membrane protection, cell wall modification, and substrate recycling. Upregulated genes were primarily enriched in linoleic acid metabolism (10 genes), pentose and glucuronate interconversions (15 genes), lysine (9 genes), and glycosaminoglycan (3 genes) degradation. Linoleic acid metabolism not only stabilizes membranes and generates signaling oxylipins (67), but also supports the synthesis of barrier compounds like cutin and suberin (9). Pentose and glucuronate interconversions support cell wall reinforcement and carbon redirection for osmoprotection, while lysine degradation recycles nutrients to fuel defense responses (68, 69). The upregulation of linoleic acid metabolism directly contributes to enhanced membrane stability by facilitating lipid remodeling and the production of oxylipins as signaling molecules, which helps reduce lipid peroxidation (Fig. 2), as lower lipid peroxidation reflects improved membrane integrity resulting from an adjusted fatty acid composition (70, 71). This transcriptomic adjustment complements the observed physiological improvements in antioxidant enzyme activities (SOD, CAT, and POD) and overall thermotolerance.

Complementing the metabolic activation, ECM symbiosis under heat stress suppressed genes in energy metabolism and core physiological processes (Fig. 7), reflecting a strategic resource reallocation. This downregulation of oxidative phosphorylation genes likely reduces ROS generation at the mitochondrial electron transport chain, which is the primary source of ROS in root tissue under heat stress (24). Such a reduction in mitochondrial ROS load could partly account for the lower MDA levels and better-preserved antioxidant enzyme activities (SOD, CAT, and POD) observed in ECM-inoculated roots under 35°C (Fig. 2). Downregulation of oxidative phosphorylation and photosynthesis likely reduces internal ROS production, while suppressed RNA polymerase activity minimizes energy-intensive transcription (72). Although this includes a reduction in some antioxidant pathways, like glutathione and flavonoids, the concurrent shift toward lipid and carbohydrate metabolism indicates a prioritized investment in membrane and osmotic protection (73). Finally, the observed downregulation of circadian rhythm genes further suggests a reorientation away from diurnal regulation toward sustained stress defense (74).

A direct comparison of the DEG compositions between the two temperature-response contrasts (25°C vs 35°C) further reveals how ECM inoculation reshapes the host transcriptional response to heat. In non-inoculated seedlings, elevated temperature triggered 3,761 DEGs in total (2,340 upregulated and 1,421 downregulated), whereas in inoculated seedlings the same thermal shift induced only 2,657 DEGs (1,351 upregulated and 1,306 downregulated), indicating that ECM colonization broadly attenuates the scale of transcriptional perturbation caused by heat stress. Of these, only 565 upregulated and 409 downregulated genes were shared between the two comparisons, demonstrating that the majority of heat-responsive genes are treatment-specific. Notably, 93 genes were upregulated by heat in non-inoculated seedlings but downregulated in inoculated seedlings under the same thermal shift, including genes encoding RNA polymerase subunits (e.g., DNA-directed RNA polymerase subunit β′) and mitochondrial NADH dehydrogenase subunits (e.g., NADH-ubiquinone oxidoreductase chain 1), suggesting that ECM actively suppresses the energy-intensive transcriptional responses that heat otherwise induces in non-inoculated plants. Conversely, 18 genes were downregulated by heat in non-inoculated seedlings but upregulated in inoculated seedlings, including pathogenesis-related protein 1, 1-aminocyclopropane-1-carboxylate oxidase, and expansin, indicating that ECM inoculation restores or activates defense and cell wall remodeling programs that are otherwise suppressed by heat stress in the absence of fungal symbiosis. Thus, ECM symbiosis implements a resource reallocation strategy at the transcriptomic level, pivoting from routine metabolic functions toward a focused defense program that prioritizes cellular integrity and minimizes internal oxidative stress, thereby conferring a robust thermotolerance advantage to the host. It is worth noting that some of the transcriptional differences observed between inoculated and non-inoculated seedlings may not reflect direct ECM-mediated regulatory effects, but could instead be an indirect consequence of the overall greater growth vigor and physiological status of inoculated plants. Differences in plant size and metabolic activity *per se* can drive broad shifts in gene expression, and this caveat should be considered when interpreting ECM-specific molecular mechanisms. Nevertheless, a direct comparison of the transcriptional responses to elevated temperature between the inoculated group (25°C vs 35°C Inoculation) and the control group (25°C vs 35°C Control) reveals that ECM inoculation fundamentally reshapes how the host transcriptome responds to heat stress. In non-inoculated seedlings, high temperature primarily induced upregulation of oxidative phosphorylation and RNA polymerase-related genes, reflecting a generalized stress response. In contrast, inoculated seedlings under the same thermal shift showed suppression of these energy-intensive pathways and instead activated protective processes, including linoleic acid metabolism and cell wall modification. This divergence indicates that *C. geophilum* inoculation does not merely amplify the plant’s default heat response, but redirects it toward a more targeted and protective transcriptional program.

### Conclusion

**Fig 9:**
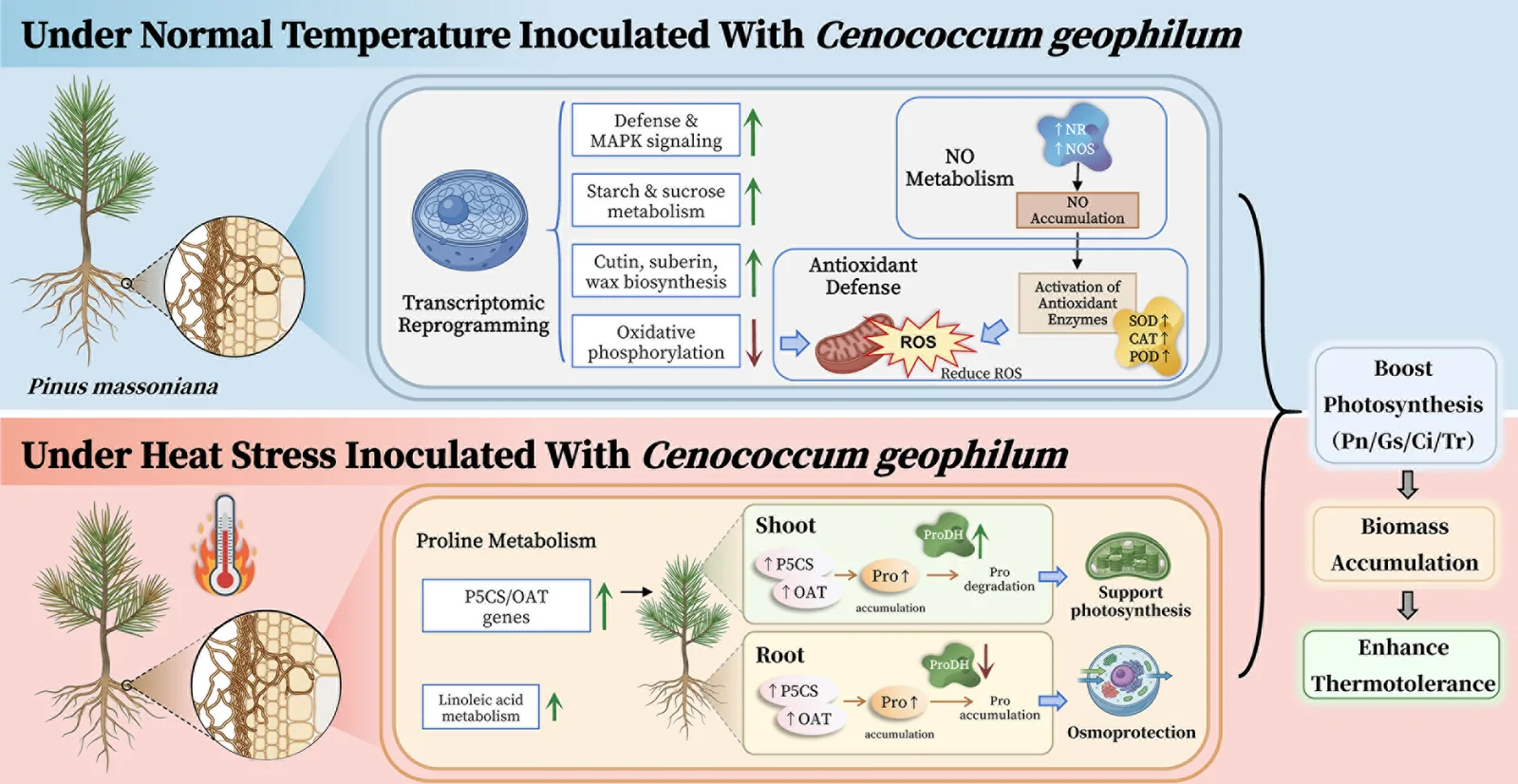
Proposed mechanistic framework for *Cenococcum geophilum*-mediated thermotolerance enhancement in *Pinus massoniana* seedlings under normal temperature (25°C, upper panel) and heat stress (35°C, lower panel). Green and red arrows indicate upregulation and downregulation of the indicated processes, respectively.

This study demonstrates that *Cenococcum geophilum* inoculation significantly enhances thermotolerance in *Pinus massoniana* seedlings through integrated physiological, biochemical, and transcriptional mechanisms. Under heat stress, ECM symbiosis promoted host biomass recovery (up to 118% in roots), elevated antioxidant enzyme activities (SOD by up to 862%), and enhanced NO accumulation (by 39–150%), effectively mitigating oxidative damage. ECM also reprogrammed proline metabolism in a tissue-specific manner to reinforce osmotic adjustment and energy balance. Transcriptomically, ECM primed host defense pathways at normal temperature and drove strategic resource reallocation under heat stress, upregulating membrane protection and carbon redistribution while suppressing energy-intensive processes to prioritize cellular integrity. These integrated mechanisms are summarized in a proposed framework (Fig. 9).

These findings establish a mechanistic framework for ECM-mediated climate resilience and highlight *C. geophilum* inoculation as an ecologically sustainable tool for enhancing forest thermal adaptability. Future research should validate these effects under field conditions, explore the genetic basis of thermotolerance variation among ecotypes, decipher ECM-induced NO signaling networks and fungal effector interactions, and integrate proteomic and metabolomic approaches to fully substantiate the functional significance of the observed transcriptional reprogramming, ultimately advancing the translation of these findings into actionable forest management strategies.

## Data Availability

The raw sequencing data generated in this study are deposited in the NCBI Short Read Archive (SRA) database under BioProject accession number PRJNA1377802, which are available under accession numbers SRR36417797–SRR36417808.

## SUPPLEMENTAL MATERIAL

Table S1 aem.02491-25-s0001.xlsx Key pathways of differentially expressed genes (DEGs) for all comparisons.

ASM does not own the copyrights to Supplemental Material that may be linked to, or accessed through, an article. The authors have granted ASM a non-exclusive, world-wide license to publish the Supplemental Material files. Please contact the corresponding author directly for reuse.
